# A Conversation with Olga Dudchenko

**DOI:** 10.1021/acscentsci.4c02010

**Published:** 2024-12-11

**Authors:** Carolyn Wilke

In the summer of 2024, researchers reported that they had sequenced genes from the 52,000-year-old DNA of a woolly mammoth. The ancient beast was unearthed in Siberia, where it had been preserved in permafrost. Freeze-drying had turned the animal’s skin to a stable glass that maintained the 3D arrangement of chromosomes.

**Figure:**
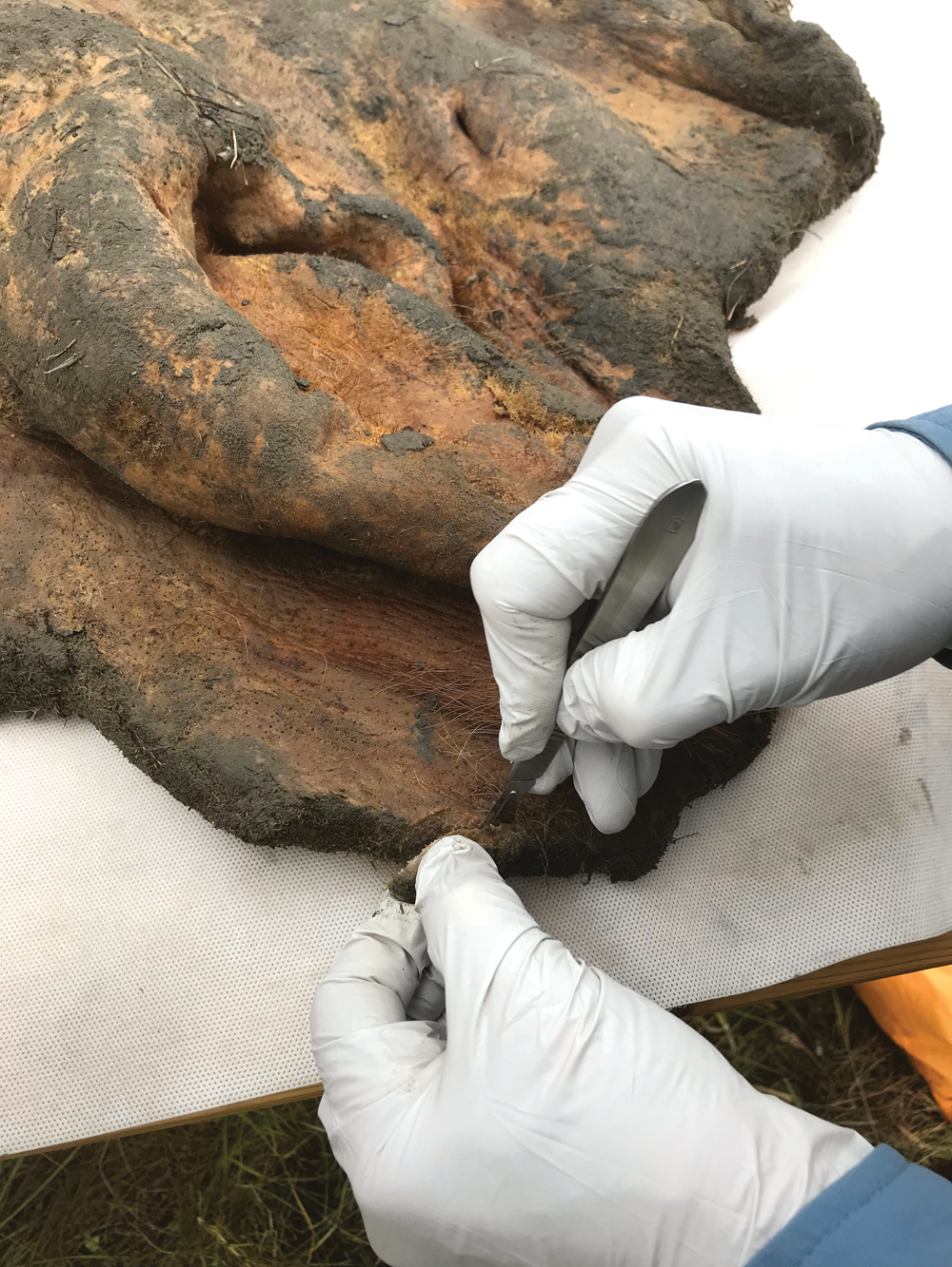
Researchers analyzed genetic material from a 52,000-year-old woolly mammoth sample that had been preserved in permafrost. The team found clues to the biology of the animal and how it differs from modern elephants by studying the 3D structure of chromosomes. Credit: Love Dalén/Stockholm University.

“What we were looking at was, in some sense, a piece of mammoth jerky,” says Olga Dudchenko, an applied physicist and mathematician working in genomics at Baylor College of Medicine. Dudchenko was one of the leaders of this 9-year project, along with collaborators at the University of Copenhagen and the University of Barcelona.

The team aimed to analyze an ancient sample using a variation of Hi-C, a high-throughput technique for examining the 3D structure of DNA. The method can illuminate the function of cells and assemble DNA sequencing data into an organism’s genome.

Using PaleoHi-C, a version adapted for ancient genetic material, the team was able to find hundreds of genes that were active in mammoths but not in elephants, and vice versa. Carolyn Wilke spoke with Dudchenko about the technique the researchers used, how it works, and its potential. This interview was edited for length and clarity.

## Why did your team investigate the 3D structure of ancient mammoth DNA?

There was a lot of discussion about whether the 3D structure of genomes can somehow be preserved through time. One of the study’s reviewers said that, before this paper, they did not think it was possible.

If you look at illustrations, even from the press release for the Nobel Prize that Svante Pääbo won for advancing the field of ancient DNA, you see that people think of ancient DNA sprinkled over the archeological or paleo site. It’s like pieces of DNA lying somewhere mixed with contaminants.

DNA is a fragile molecule. After an organism dies, those long polymers start fragmenting. Nucleases come and cut the DNA, and chemical reactions like hydrolysis will break the DNA. It’s not unreasonable to expect that everything will kind of float apart because of diffusion. But we were curious. Is there any possibility that maybe in some circumstances somehow the DNA pieces will still remain in their relative positions?

We launched a search party. For 5 years, we were more or less searching in vain. Then in our fifth year, we found a sample that looked promising—the woolly mammoth.

## Why would we want to examine the 3D structure of DNA using a method such as Hi-C?

If you take the DNA out of any one cell in your body and stretch it out, that polymer molecule is about 2 m in length. Somehow those long polymer noodles are crammed inside a nucleus which is like 6 μm in diameter. Spoiler alert: the way that DNA is crammed in is not random. There is a functional significance to how DNA is folded. Most of the 2 m of DNA are more or less identical. However, the cells do very different things. It can be useful to think of DNA as an origami print of an instruction manual. Depending on how you fold it, you see slightly different instructions. There’s a relationship between the way that cells operate and the way their genomes are folded.

The most natural way to figure [how DNA is folded in a cell] is to look at DNA under a microscope. People would label pieces of DNA with different colors and see where they come together. This works well, except there’s so many positions. So Erez Lieberman Aiden and his collaborators came up [with Hi-C] to read off information about who’s near who in 3D at high throughput. [Aiden is at Baylor College of Medicine and is one of the paper’s coauthors.—Ed.]

## How does Hi-C work to reveal this spatial information about DNA?

We cut the DNA molecules when they are still in their original 3D position. Then we ligate them. The ligase is basically an enzyme that [if] it sees two hanging ends, it will glue them together. It doesn’t know who was the original neighbor. Sometimes pieces will ligate back to their neighbors [in the linear sequence], but sometimes a ligase will just grab something that’s nearby in 3D and fuse them together into a 1D chimera. We can read the sequence off as a single unit.

You’re reading off a piece of DNA that comes from somewhere and another piece of DNA that was near it in 3D. The Hi-C experiment basically tells us which positions of the genome were nearby in 3D.

We assembled [genomes of] the Asian elephant and the African elephant, and then we used some of the information about elephantids when assembling the mammoth [genome]. But we used it in a very different way than people have previously.

People would take the sequences from mammoths and find the corresponding place in the elephant genome. The overall context, the structure of whole chromosomes, was fully reliant on the elephant. For example, people did not know how many chromosomes the mammoth had. They could just guess that it was the same as the elephant. We don’t have to guess. We’ve now confirmed that the context is quite similar.

Inherently, the procedure that we put forward should work, even if there is no close relative like the elephants, as long as you get enough data.

It proved that knowing how the genome folds actually can be very useful to help with assembling the genome from fragments of DNA.

**Figure:**
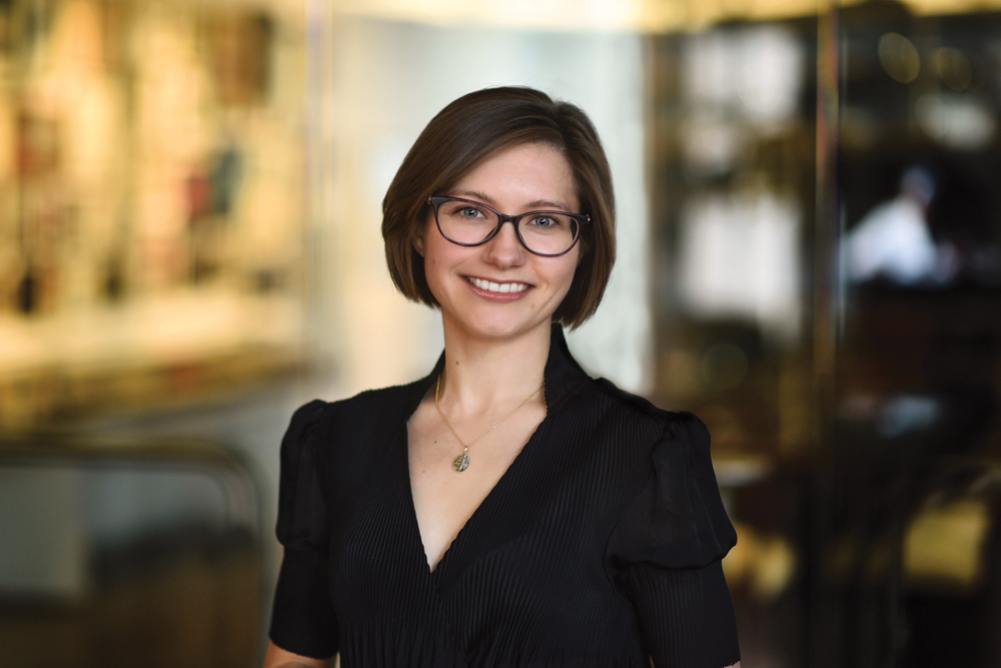
Credit: Baylor College of Medicine.

## Why is this helpful for assembling genomes?

We typically sequence the genome in pieces. You read off maybe 100 base pairs at a time. But those long polymers are on the scale of 100 million base pairs. You need somehow to figure out how they overlap with each other to try to build the full sequence.

The problem is real genomes contain repeat sequences. The classical analogy is a jigsaw puzzle. There are these superfrustrating parts—like a blue sky. There are no features that span across individual pieces. But sometimes you have border pieces, so there is some spatial information that has nothing to do with the picture itself.

In genome assembly, having information about how a genome folds in 3D is quite useful. The content is not even important, but it provides some constraints that reduce the complexity of the problem.

We have this project called the DNA Zoo consortium, where we basically put these kinds of methods to work to assemble species and release [their genomes] as an open-source resource. It’s proven useful for the conservation community because often they don’t have resources to generate genomes.

## Why did you need to adapt Hi-C to create PaleoHi-C to work on ancient samples?

There’s just very little material. Usually in a modern sample you would extract nuclei. In paleo samples, there is no guarantee that there will be any intact nuclei. We had to come up with a way to work with dirty samples or just pieces of nuclei and what’s called chromatin, chunks of DNA associated with proteins.

Also, we usually amplify the DNA to make sure we can read off as much information as we can. But ancient DNA has a lot of damage. So you need to adapt the [amplification] reactions using special polymerases to step through the damage in ancient DNA. Those adjustments were informed by the ancient DNA sequencing field.

## What did you learn from studying the mammoth’s DNA?

The way that genomes fold relates to what the cell does. That means that we can use this information to ask questions about whether a particular gene was active or not.

We created a first-of-its-kind map of gene activity in an ancient sample and compared it to the similarly generated map of gene activity in elephant skin. As you would expect, they were fairly similar.

So where would the few differences be? They actually proved to be associated with genes that are known to be associated with hair follicle development and hair maintenance. Modern elephants are more or less bald. People are not sure what made woolly mammoths woolly, which is of interest to companies like Colossal Biosciences that are trying to make something akin to a woolly elephant as a proxy for the mammoth.

## What other questions about ancient life could PaleoHi-C be used to study?

Ultimately, we’re curious about previous species because they tell us stories about adaptation. The mammoth had to adapt to a changing environment. We can see the traces of those adaptations in its genome. We can learn from that.

I certainly hope that there will be many more species for which we will be able to directly read off the information about what happened to them—not have to be confined to interpreting their stories by reading the genomes of modern species. We can directly see and we can test our ideas about how the evolution of particular species went about.

I expect that there’s going to be a plethora of studies of this kind—looking at new samples and old samples that have never been explored for this kind of information before—and that people will come up with really exciting ways to interpret and tell stories from it.

## Carolyn Wilke is a freelance contributor to

Chemical & Engineering News, *the independent news outlet of the American Chemical Society*.

